# Supplementary material for: A microfluidics platform for combinatorial drug screening on cancer biopsies
Source: Nat Commun. 2018 Jun 22;9:2434. doi: 10.1038/s41467-018-04919-w (PMC6015045; doi:10.1038/s41467-018-04919-w)
Supplement: Supplementary file 1 — Supplementary Information [file 41467_2018_4919_MOESM1_ESM.pdf]

A microfluidics platform for combinatorial drug screening on cancer biopsies

Supplementary Information

Federica Eduati[1,2,†,#], Ramesh Utharala[2,†], Dharanija Madhavan[2], Ulf Peter Neumann[3,4], Thomas Longerich[5,+], Thorsten Cramer[4,6], Julio Saez-Rodriguez[1,7,*], Christoph A. Merten[2,*]

[1] European Molecular Biology Laboratory, European Bioinformatics Institute (EMBL-EBI), Wellcome Trust Genome Campus, Hinxton CB10 1SD, UK

[2] European Molecular Biology Laboratory (EMBL), Genome Biology Unit, Meyerhofstrasse 1, 69117 Heidelberg, Germany

[3] Department of Surgery, RWTH University Hospital, 52057 Aachen, Germany

[4] ESCAM – European Surgery Center Aachen Maastricht

[5] Institute of Pathology, RWTH University Hospital, 52057 Aachen, Germany

[6] Molecular Tumor Biology, Department Surgery, RWTH University Hospital, 52057 Aachen, Germany

[7] Joint Research Centre for Computational Biomedicine (JRC-COMBINE), RWTH Aachen University, Faculty of Medicine, 52057 Aachen, Germany

†co-first authors

*co-corresponding authors: saezrodriguez@gmail.com, merten@embl.de

[#] current affiliation: Department of Biomedical Engineering, Eindhoven University of Technology, 5600MB Eindhoven, The Netherlands

[+] current affiliation: Institute of Pathology, University Hospital Heidelberg, 69120 Heidelberg, Germany

Supplementary Figures

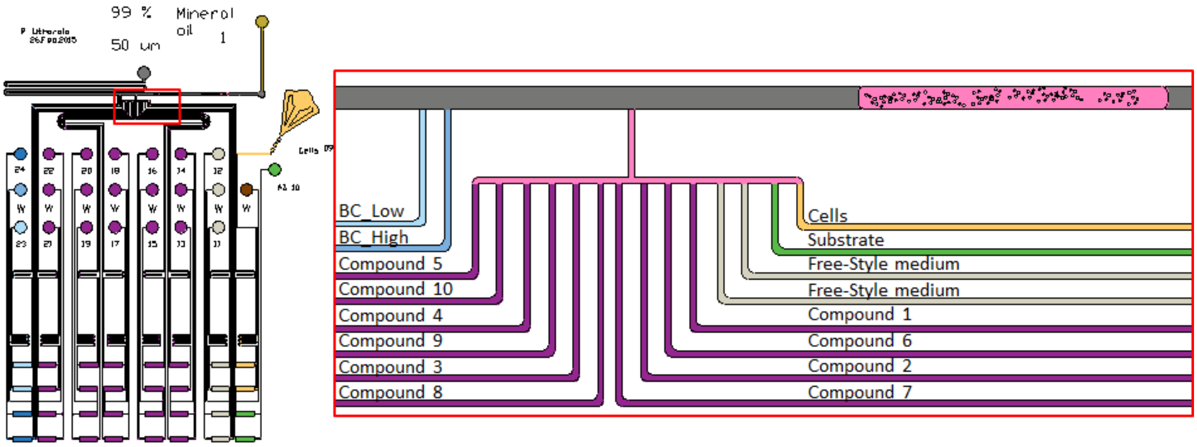

Supplementary Figure 1. Detailed schematic of the chip design. Different colors used to denote compounds (purple), barcodes (blue), cells (orange), substrate (green), and medium (grey). Combinatorial mixtures generated containing cells, substrate, medium, and compounds. The zoom-in (red colored rectangle) shows the path of each liquid and a plug containing assay components with cells.

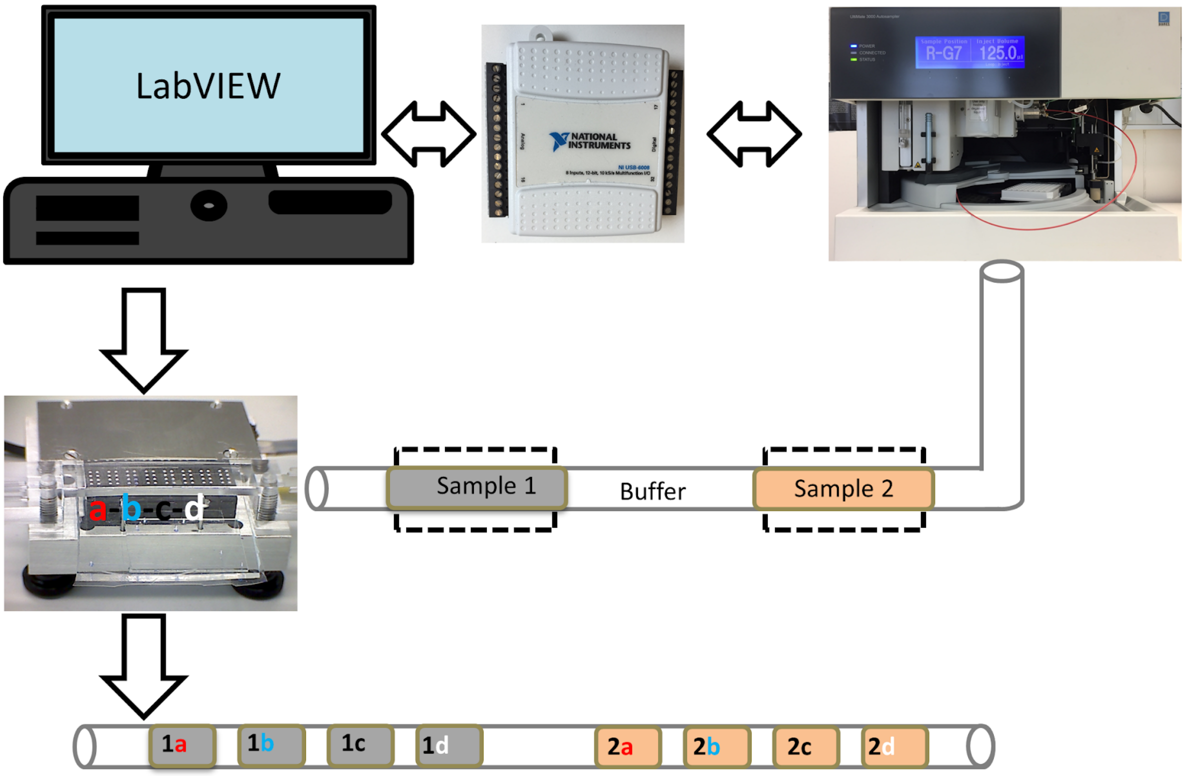

Supplementary Figure 2: Integration of an autosampler. To increase the maximum number of sample combinations, a Dionex 3000SL autosampler was integrated into the microfluidic platform. This device aspirates samples from up to three microtiter plates (96-well or 384-well format) and injects them into a target tubing. All samples are spaced out by miscible buffer and moved forward using an external Syringe pump. To overcome dispersion, operation of the autosampler and actuation of the Braille valves (controlling the inlet to which the target tubing is connected) are synchronized. This way beginning and end of each sample from the microtiter plate is sent to the waste, while the centre part (showing constant concentration) is mixed with different samples injected directly into the Braille display chip.

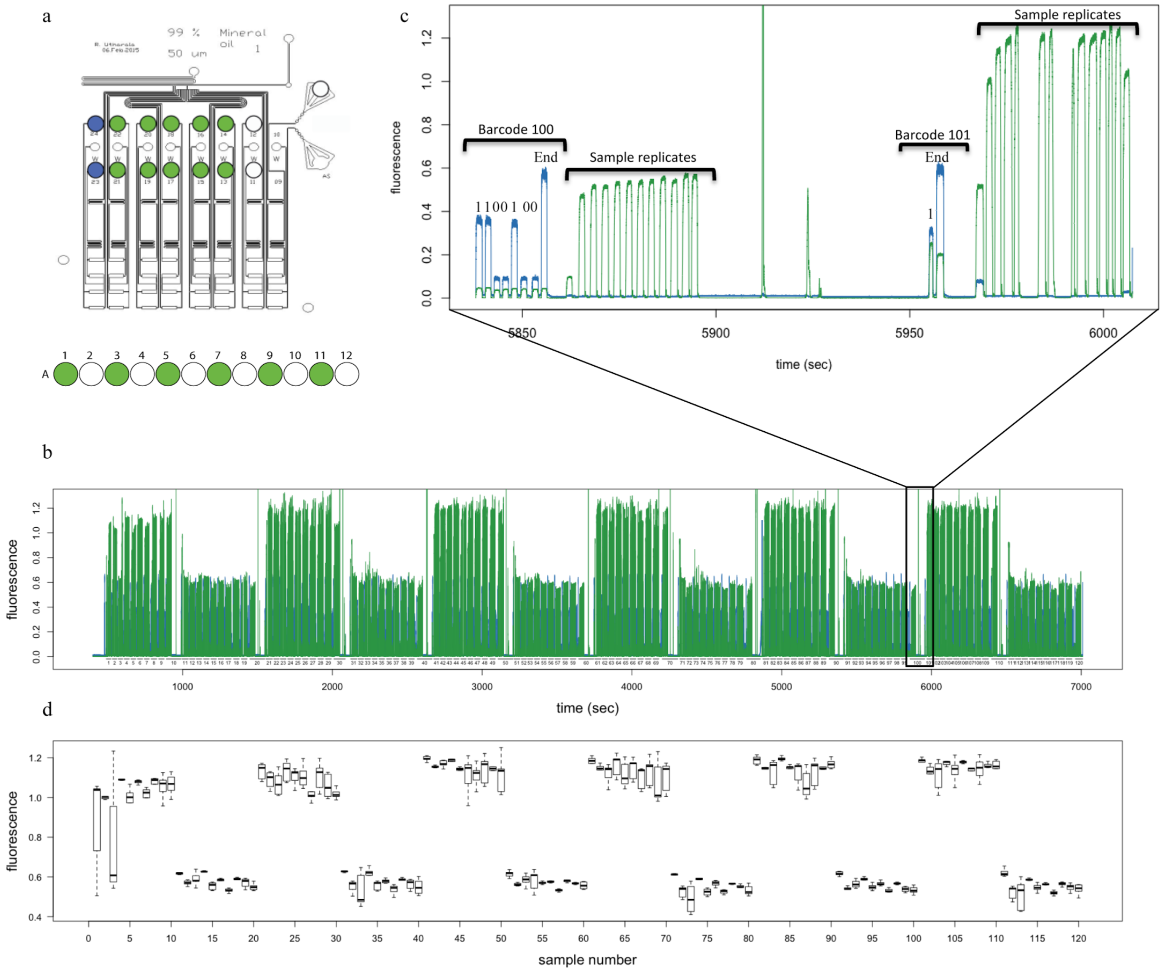

Supplementary Figure 3: Fluorescence data of sample mixtures containing reagents coming from the autosampler and the Braille display chip. (a) Samples containing 100 µM Fluorescein (green dots) were injected directly into the Braille display. In parallel, samples containing 100 µM Fluorescein (green dots) or buffer (white dots) were arrayed alternatingly within a row of a 96-well plate. (b) Subsequently all possible combinations of two fluorescein samples (high intensity peaks) or fluorescein and buffer samples (low intensity peaks) were generated. To mimic four component mixtures as used for screening, all plugs were generated while co-injecting buffer from two additional inlets of the Braille display (white dots in (a)). Samples were separated by barcodes made of binary concentrations of cascade blue (injected directly into the Braille display chip; blue dots in (a); signas shown in blue in (b,c)). Barcodes are designed such that all multiples of ten are generated as complete numbers, whereas all other barcodes just consist of numbers ranging from 1-9 (e.g. 100 corresponds to 1100100, whereas 101 corresponds to 1 as shown in (c)). (d) Subsequent data analysis confirmed the expected fluorescence intensities of the plugs (high when injecting fluorescein from the autosampler and low when injecting buffer). The total number of plugs (including barcodes) in this experiment was 1934. In all boxplots the horizontal line represents the median, the box the interquartile range and the whiskers extend to the most extreme data point which is no more than 1.5 times the length of the box away from the box.

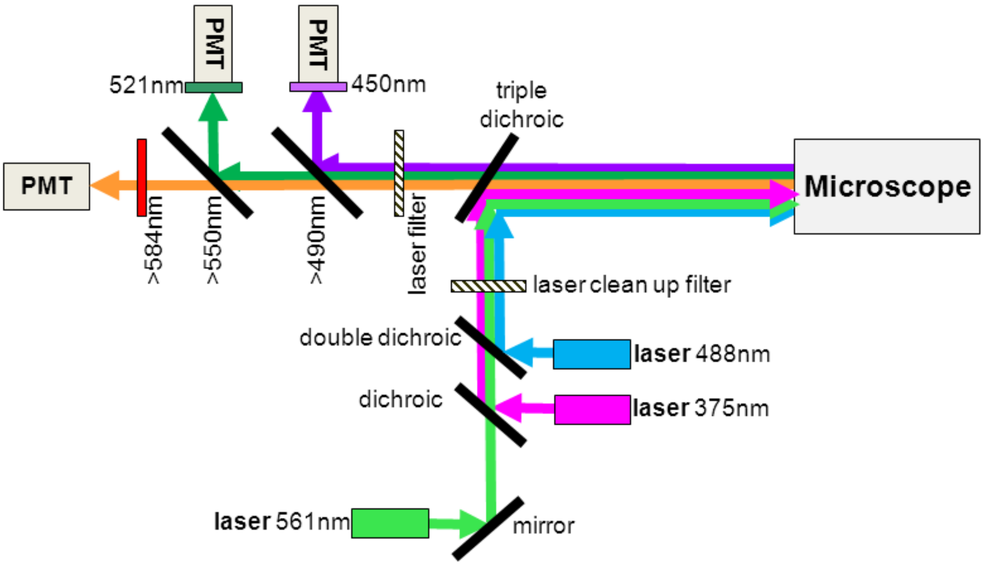

Supplementary Figure 4: Optical setup of the microfluidic workstation. Multiplexed assays are enabled by three different excitation lasers (488nm, 375nm, 561nm) and three separate emission channels (584nm, 521nm, 450nm).

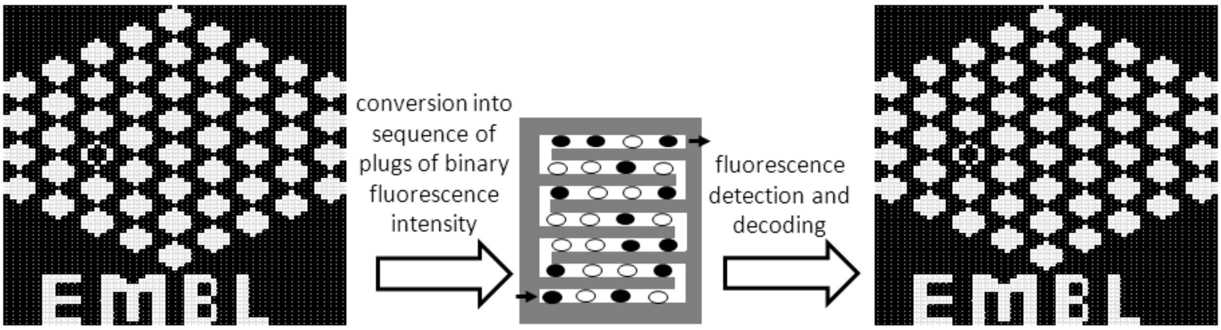

Supplementary Figure 5: Barcoding efficiency as demonstrated by transmitting image information. A simplified 2808 pixel EMBL logo was converted into a binary plug sequence and subsequently assembled *de novo*. Throughout the entire process no information was lost.

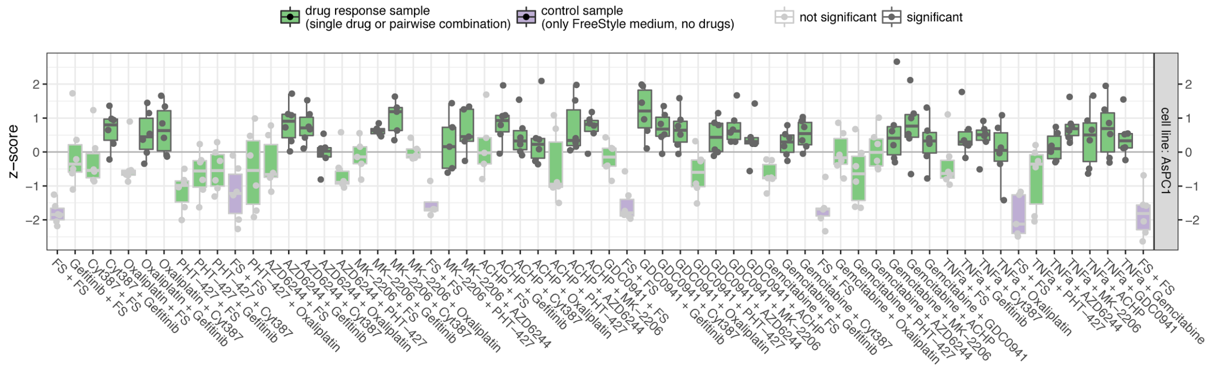

Supplementary Figure 6: Boxplot for data of AsPC1 cells. Boxplot of the sequence of samples across multiple replicates for AsPC1 cell line (z-scores of caspase-3 activity) with control conditions in purple and conditions which are not significantly (Wilcoxon rank sum test; p-value < 0.05) better than the control with light grey borders and dots. Boxplot are computed considering 6 runs, where for each run sample value is mediated across ~10 replicates. In all boxplots the horizontal line represents the median, the box the interquartile range and the whiskers extend to the most extreme data point which is no more than 1.5 times the length of the box away from the box.

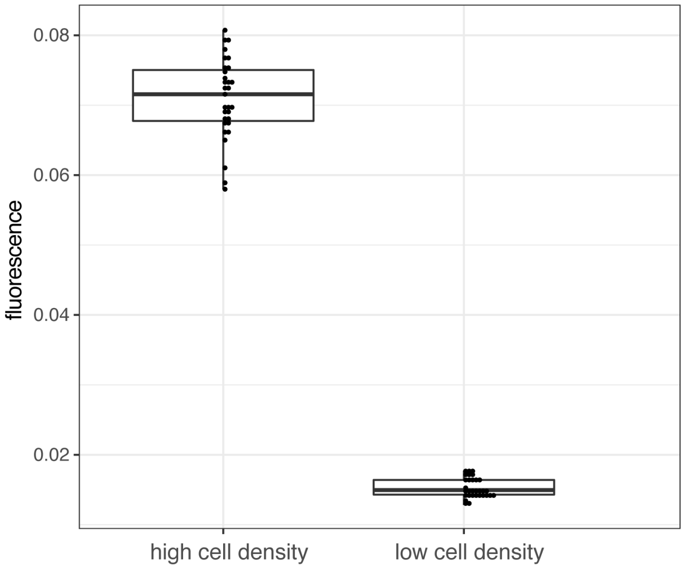

Supplementary Figure 7: Variability of cell occupancy. Boxplot of fluorescence intensity measured for the conditions with high (2*10^5 cells/ml) and low (0.5*10^5 cells/ml) cell density show clear separation between the 2 conditions. In all boxplots the horizontal line represents the median, the box the interquartile range and the whiskers extend to the most extreme data point which is no more than 1.5 times the length of the box away from the box.

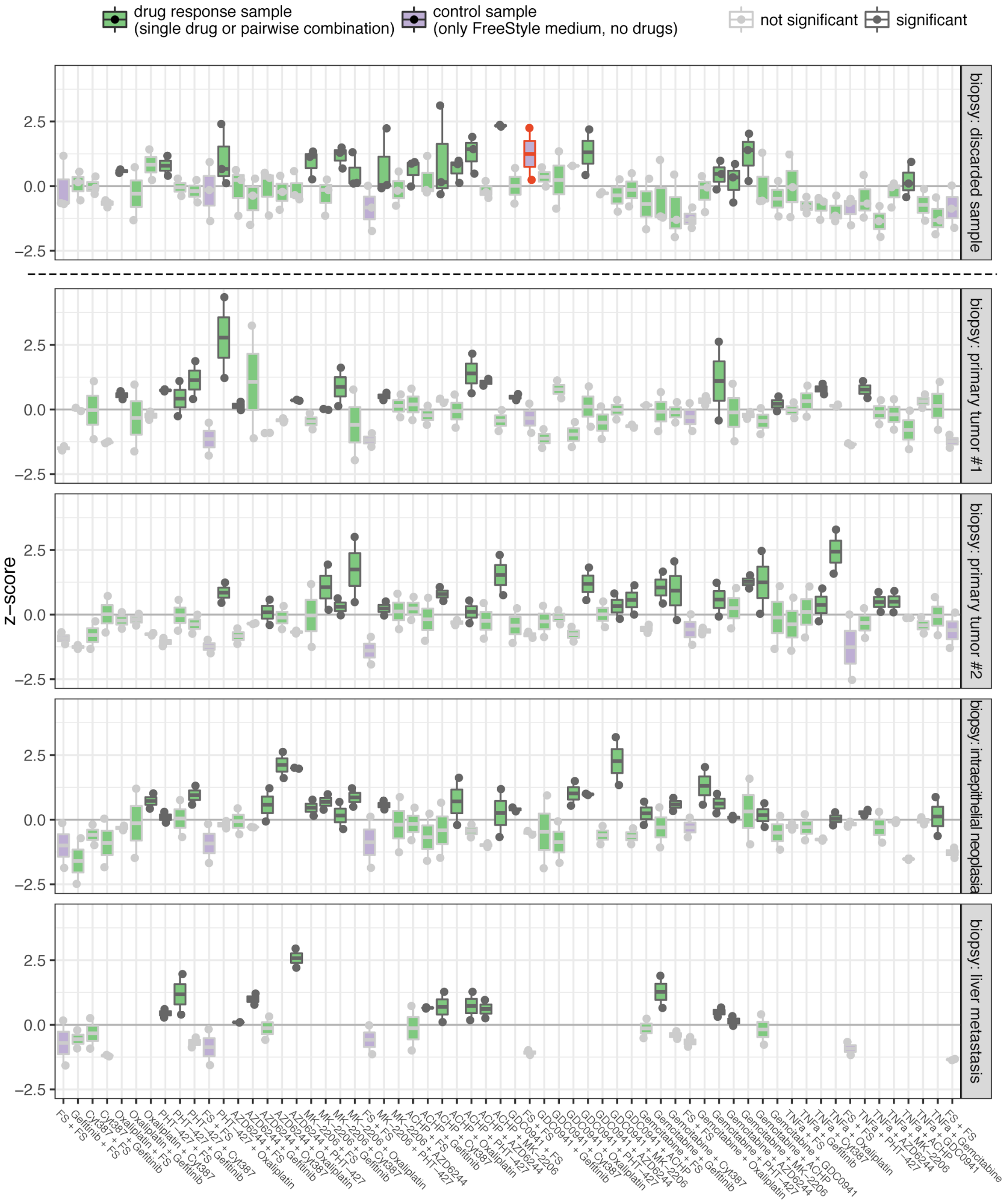

Supplementary Figure 8: Boxplot for patient samples data. Boxplot of the sequence of samples across multiple replicates for each patient (z-scores of caspase-3 activity) with control conditions in purple and conditions which are not significantly (Wilcoxon rank sum test; p-value < 0.05) better than the control with light grey borders and dots. The sample in the top panel was discarded from further analysis due to irregularity in the control signal (condition marked in red). In all boxplots the horizontal line represents the median, the box the interquartile range and the whiskers extend to the most extreme data point which is no more than 1.5 times the length of the box away from the box.

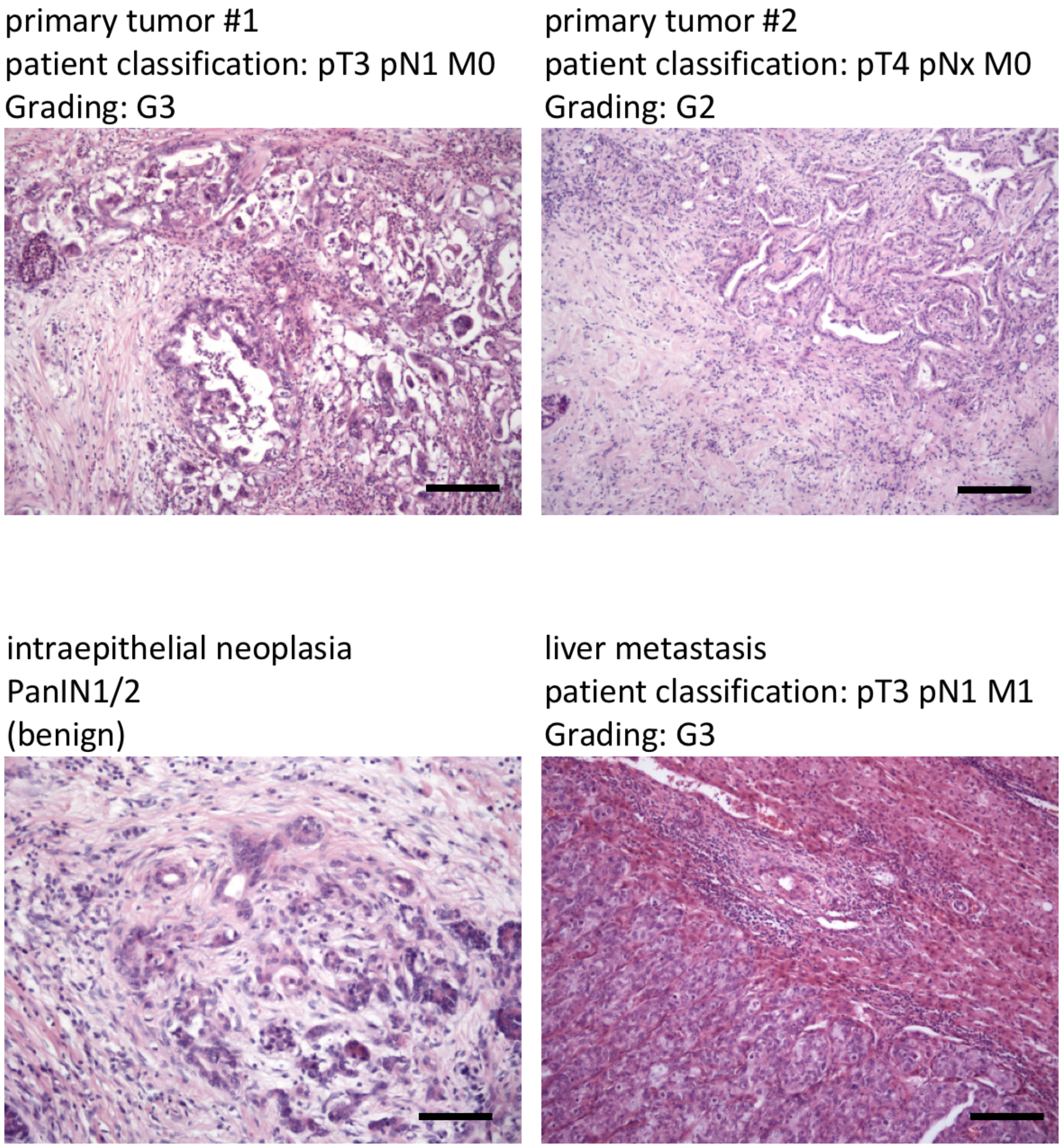

Supplementary Figure 9: Tissue sections and clinical data of the patient biopsies used in the current study. All tissue slices were stained with hematoxylin and eosin to facilitate visualization. Magnification 100x, scale bars represent 50 µm.

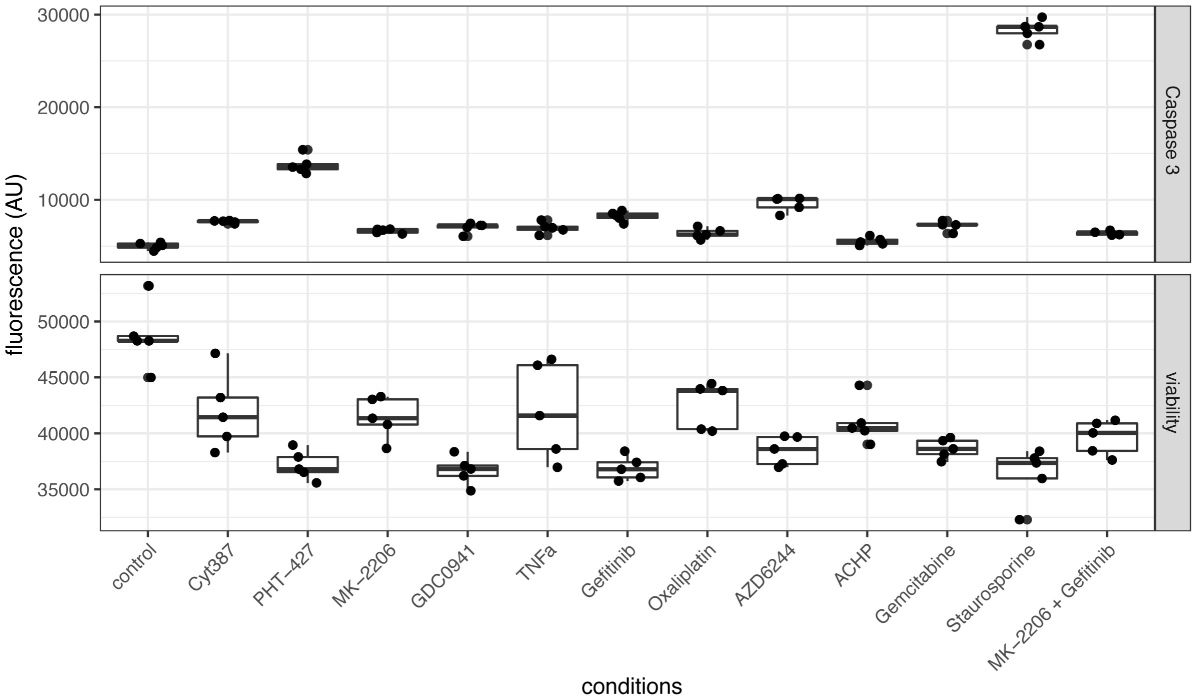

Supplementary Figure 10: Comparison of Caspase 3 and viability assays. Tissue culture experiments comparing the Caspase 3 assay ((Z-DEVD)2-R110) with a viability assay (PrestoBlue). BxPC3 cells were plated in a 96 well plate and Caspase 3 activity and cell viability were tested after perturbation with 13 different conditions (i.e. control condition with no drugs, all 10 single drugs used in the screening, one drug combination and Staurosporine as positive control) with 5 replicates each. Data measured after 16 hours of incubation with or without drugs are shown in the boxplot for Caspase 3 assay (top panel) and cell viability assay (bottom panel). Data show a strong anti-correlation between measured apoptosis and viability (Spearman correlation = -0.73, p-value = 0.006 computed considering median values across replicates for each of the 13 tested conditions) suggesting that the Caspase 3 assay is a good early marker of cell viability. In all boxplots the horizontal line represents the median, the box the interquartile range and the whiskers extend to the most extreme data point which is no more than 1.5 times the length of the box away from the box.

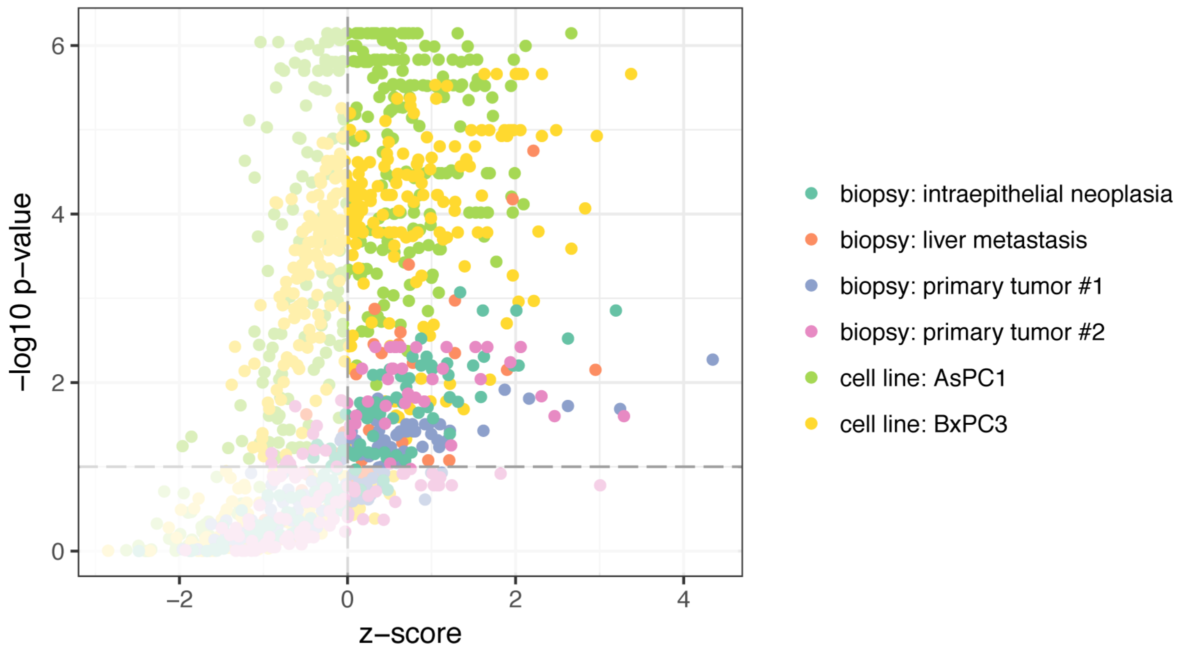

Supplementary Figure 11: Significance and z-scores for all data shown in the manuscript. The z-score for each patient/cell-line are shown for each condition and each run, along with the corresponding p-value assessing the statistical difference with respect to the control condition. Thresholds are set to z-score > 0 and FDR-corrected p-value (from Wilcoxon rank sum test) < 0.1. The P-value can be interpreted as a measure of the variability of the replicate, revealing that, as expected, cell lines showed a lower variability with respect to patient samples probably due to the higher heterogeneity of the cells in patient samples. However both cell lines and patient samples showed strong and robust response to specific drug combinations.

Supplementary Notes

Supplementary Note 1: Cross-contamination between plugs

A certain extent of cross-contamination between plugs is due to the dead volume in the chip, causing a leftover from the previous reagents that can be cleared with the first one or two plugs of each experimental condition. Each experimental condition is preceded by a blue barcode, therefore the intensity of the blue signal is an indication of the cross-contamination. The recorded signals for BxPC3 cell lines clearly show that there is about 10.7% cross-contamination in the first plug for any given condition (as % of first peaks falling in the higher 5% of the overall population of blue peaks; % is reported as median value across the 6 runs), 1.6% in the second plug and ~0% from the third plug onwards.

Supplementary Note 2: Summary of costs per experiment

Here below (Supplementary Table 1) a summary of costs for all material (chemicals, syringes, tubings, chip fabrication, ...) used in one experiment as purchased in €. Costs have been converted to $ using exchange rate of 1.14 and rounded up to the next integer.

Supplementary Table 1.

	Price (€)	Price ($)	Note
Caspase 3 assay	27 €	~ 31 $	Bought in bulk from Biomol at 80 € for 1 mg. 0.33 mg per experiment used.
Compounds	11 €	~ 13 $	See detailed costs in table below
Fluorescent dyes	13 €	~ 15 $	~7 € for Alexa Fluor 594 (< 0.1 mg per experiment, costs 362 € for 5 mg) + ~ 6 € for Cascade Blue (< 0.195 mg per experiment, costs 305 € for 10 mg)
Medium	12 €	~ 14 $	Based on 1 L FreeStyle medium bottle used for ~13 experiments (5 ml x 15 syringes).
Syringes	4 €	~ 5 $	18 syringes used
Tubing	14 €	~ 16 $	Storage of plugs (~ 8 €) and Syringes (~ 6 €) Tubing for experiment is reusable
Chip	3 €	~ 4 $	Chip fabrication (PDMS and crosslinker costs), mold fabrication (photoresist, developer and silicon wafer; one mold used for ~ 200 chips)
Others	~ 2-3 €	~ 3-4 $	Buffer components, Xanthan Gum, Pluronic, needles, oils (FC-40 + MO)...

Detailed costs for compounds:

	Vendor	Catalog No.	Price [€]	Amount [mg]	No. of Experiments *	Cost per experiment [€]
Cyt387	Selleck	S2219	167	10	121	1.38
PHT-427	Selleck	S1556	68	10	122	0.56
MK-2206	Selleck	S1078	108	5	52	2.08
ACHP	Tocris	4547	255	10	134	1.90
Gefitinib	Selleck	S1025	144	100	448	0.32
Gemcitabine	Selleck	S1149	68	25	417	0.16
Oxaliplatin	Selleck	S1224	68	50	629	0.11
AZD6244	Selleck	S1008	88	50	546	0.16
GDC-0941	Selleck	S1065	117	5	49	2.39
TNF-alpha	Invitrogen	PHC3015	81.75	0.01	50	1.64

* number of experiments are computed by considering how many 10 µL (20 µL for TNF) aliquots can be prepared at stock concentration of 20 mM (10 µg/ml for TNF). This is an underestimate since only half of the volume of each aliquot (5 µl for drugs and 10 µl for TNF) is actually used in each experiment.

Supplementary Note 3: Barcoding plugs

The barcodes are based on sequences of plugs with binary (high = 48 µM /low = 16µM) concentrations of the blue fluorescent dye cascade blue (Supplementary Movie 3). Each plug is generated by mixing 4 aqueous phases in total, each injected at a flow rate of 500 µl/h. During the generation of a barcode the dye is hence diluted 4-fold, resulting in 12 µM (high) or 4 µM (low) concentrations. However, for the end of barcode signal both dyes are injected in parallel, meaning that the final concentrations is (48 µM + 16 µm)/4 = 16 µM. Therefore, the end of barcode signal shows an even higher fluorescence intensity than a high fluorescence digit of the barcode itself.

Supplementary Note 4: Ab/adsorption of drugs in PDMS

The ab/adsorption of small molecules in our chip is negligible given that we continuously inject the reagents and anyways discard the first 1-2 plugs from analysis which, in case there is any loss, should be affected most. This is also supported by the fact that a) the results from the microfluidic screens could be validated for cell lines in ordinary plastic culture dishes and b) the intensity of the first barcodes (which should be affected most by potential reagent loss) is not lower than that of the last ones.

Supplementary Note 5: Diffusion of compounds in mineral oil

To rule out that drugs diffuse into the mineral oil spacers we performed a comprehensive series of experiments: We dissolved 100 µM PHT-427, a particularly hydrophobic drug (logP = 7.4) used in our screens, in 50 µl of water (control sample). In parallel, we set up a second sample and additionally added 50 µl of FC40 50 µl of mineral oil and 0.5% PFO in FC-40 (test sample). Both samples were incubated at 37°C for 8 hours under constant rotation. Subsequently the aqueous phases were recovered and analyzed by Ultra-Performance Liquid Chromatography (UPLC, 254 nm readout signal). For both samples, the area under the curve of the compound peak was comparable, with only minor loss of PHT-427 for the test sample (AUC = 51.1 compared to 61.7 for the control sample). We then also repeated the experiments with Afatinib (log P = 3.7), a breast cancer drug whose effect can also be easily assessed in cell culture (in contrast to PHT-427, which did not show a significant effect when applied as a single drug). After the UPLC results confirmed a similar trend as observed for PHT-427 (AUC = 326.95 for the test sample and 393.95 for the control sample) we also used the aqueous phases to assess their efficiency in promoting apoptosis and reducing viability. To do so, plugs containing 5uM of afatinib diluted in media were generated using the braille display in a manner similar to the actual experiments. After incubating for 6 hours at 37°C the plugs were flushed out. The aqueous phase corresponding to the media with Afatinib was then applied to BT474 cells (breast cancer cell line sensitive to afatinib). Additionally, 5uM afatinib in media simultaneously incubated at 37°C was used as a positive control. After 12h viability was read by Presto blue assay. Viability (Supplementary Fig. 12) was found to be comparably reduced in both positive control (PC) and plugs treated cells, demonstrating that the drug (a) did not leak from the aqueous plug into either of the oil phases, and (b) potency of drug was not affected.

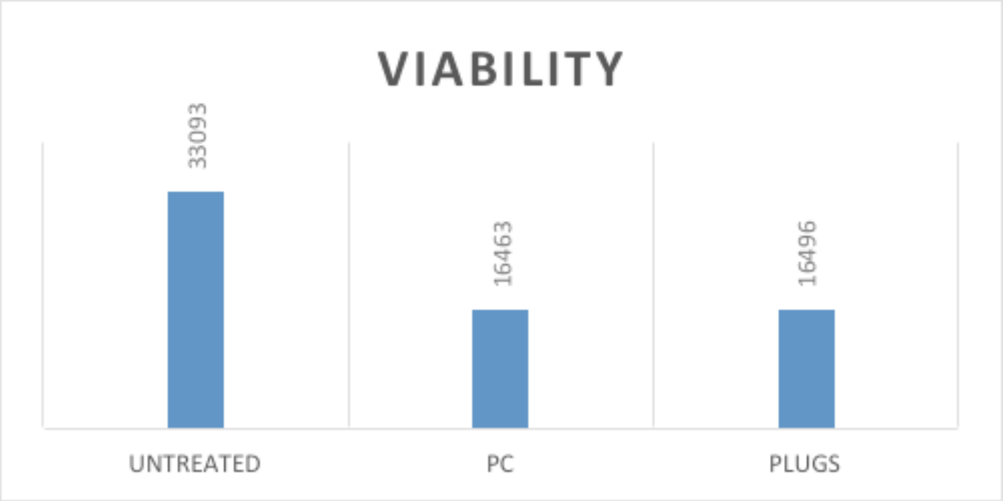

Supplementary Figure 12.
