# Supplementary material for: A microfluidics platform for combinatorial drug screening on cancer biopsies
Source: Nat Commun. 2018 Jun 22;9:2434. doi: 10.1038/s41467-018-04919-w (PMC6015045; doi:10.1038/s41467-018-04919-w)
Supplement: Supplementary file 3 — Description of Additional Supplementary Files [file 41467_2018_4919_MOESM3_ESM.pdf]

Description of Additional Supplementary Files

File Name: Supplementary Movie 1
Description: Braille valve in action. A cross-section of a microfluidic chip made entirely of PDMS (bonded to a PDMS membrane) is mounted on top of a Braille display. Upon actuation of the pins, the channels (showing rectangular cross sections) can be closed off completely.

File Name: Supplementary Movie 2
Description: Mineral oil spacing. At the outlet of the microfluidic chip, mineral oil is injected through a perpendicular channel (see design in Fig 1B). This results in regular spacing of the sample plugs within the fluorinated carrier phase.

File Name: Supplementary Movie 3
Description: Generation of barcodes. General principle of sample barcoding. Binary digits in form of small plugs with two different concentrations of a fluorescence dye (high/low) are generated in between the larger sample plugs.
